# Placental imprinting of *SLC22A3* in the *IGF2R* imprinted domain is conserved in therian mammals

**DOI:** 10.1186/s13072-022-00465-4

**Published:** 2022-08-27

**Authors:** Teruhito Ishihara, Oliver W. Griffith, Shunsuke Suzuki, Marilyn B. Renfree

**Affiliations:** 1 School of BioSciences, The University of Melbourne, Melbourne, VIC 3010 Australia; 2 Department of Biological Sciences, Macquarie University, Sydney, NSW 2109 Australia; 3 Department of Agricultural and Life Sciences, Faculty of Agriculture, Shinshu University, Nagano, 399-4598 Japan

## Abstract

**Background:**

The eutherian *IGF2R* imprinted domain is regulated by an antisense long non-coding RNA, *Airn*, which is expressed from a differentially methylated region (DMR) in mice. *Airn* silences two neighbouring genes, *Solute carrier family 22 member 2* (*Slc22a2)* and *Slc22a3*, to establish the *Igf2r* imprinted domain in the mouse placenta. Marsupials also have an antisense non-coding RNA, *ALID*, expressed from a DMR, although the exact function of *ALID* is currently unknown. The eutherian *IGF2R* DMR is located in intron 2, while the marsupial *IGF2R* DMR is located in intron 12, but it is not yet known whether the adjacent genes *SLC22A2* and/or *SLC22A3* are also imprinted in the marsupial lineage. In this study, the imprinting status of marsupial *SLC22A2* and *SLC22A3* in the *IGF2R* imprinted domain in the chorio-vitelline placenta was examined in a marsupial, the tammar wallaby.

**Results:**

In the tammar placenta, *SLC22A3* but not *SLC22A2* was imprinted. Tammar *SLC22A3* imprinting was evident in placental tissues but not in the other tissues examined in this study. A putative promoter of *SLC22A3* lacked DNA methylation, suggesting that this gene is not directly silenced by a DMR on its promoter as seen in the mouse. Based on immunofluorescence, we confirmed that the tammar SLC22A3 is localised in the endodermal cell layer of the tammar placenta where nutrient trafficking occurs.

**Conclusions:**

Since *SLC22A3* is imprinted in the tammar placenta, we conclude that this placental imprinting of *SLC22A3* has been positively selected after the marsupial and eutherian split because of the differences in the DMR location. Since SLC22A3 is known to act as a transporter molecule for nutrient transfer in the eutherian placenta, we suggest it was strongly selected to control the balance between supply and demand of nutrients in marsupial as it does in eutherian placentas.

**Supplementary Information:**

The online version contains supplementary material available at 10.1186/s13072-022-00465-4.

## Background

Genomic imprinting is a complex epigenetic process that leads to expression of a subset of genes in a parent-of-origin specific way [1, 2]. Amongst vertebrates, this phenomenon has been found in therian mammals (eutherians and marsupials), but there is no evidence for imprinting so far in monotreme mammals or non-mammalian vertebrates [3–6]. Since many imprinted genes function in the mammalian placenta, genomic imprinting is thought to have evolved in concert with mammalian placentation [7–12]. In this context, the ‘supply and demand’ theory suggests that imprinted genes in the placenta evolved to regulate the balance of nutritional interactions between mother and the fetus carrying father’s genes by controlling the supply of nutrients [13]. However, postnatal imprinting has been characterised in both marsupials and eutherians [14–17], so imprinting is involved not only in placentation, but also in postnatal growth and development. The question remains as to how and why genomic imprinting evolved only in therian mammals amongst vertebrates. As imprinting must have evolved in the common ancestor of therian mammals, by comparing the characteristics of imprinting between marsupials and eutherians, we may be able to identify ancestral features of imprinting and understand how it evolved.

Imprinted genes of eutherians tend to be clustered in the genome [18, 19]. The grouping of imprinted genes within clusters allows sharing of common regulatory elements such as non-coding RNAs and differentially methylated regions (DMRs). When these regulatory elements control the imprinting status of clustered genes, they are known as imprinting control regions (ICRs). In eutherians, one of the best characterised imprinting clusters is the *Insulin-like growth factor 2 receptor (IGF2R)* gene locus [20–26]. In mice, the *Igf2r* gene is a paternally imprinted (maternally expressed) gene, and its imprinting is regulated by an intrinsic CpG island, which is a DMR, and the antisense long non-coding (lnc) RNA, *Airn* [23, 25–28]. The DMR is established during gametogenesis and has differential epigenetic modifications between gametes [29, 30]. It acts as a promoter of the lncRNA, *Airn* [27]. *Airn* is a maternally imprinted (paternally expressed) gene and silences *Igf2r* expression on the paternal genome by transcriptional overlap [23]. This lncRNA also regulates the imprinting of a 10 + Mb region that includes two neighbouring genes in the placenta, the *solute carrier family 22 members 2 and 3* (*Slc22a2 and Slc22a3)* [22, 28, 31] as well as seven distal genes more than 2 Mb away from the *Igf2r* locus [32]. *Airn* establishes epigenetic silencing of *Slc22a3* by recruiting a histone modification enzyme, euchromatin histone methyltransferase 2 (EHMT2, also known as G9a) [31]. Although the exact mechanism by which *Airn* inactivates the paternal *Slc22a2* is currently unknown, *Airn* may recruit histone 3 lysine 27 trimethylation (H3K27me3) and the polycomb repressive complex 2 (PRC2) to the gene locus [22]. Therefore, the DMR at the intrinsic CpG Island which controls *Airn* expression is the ICR of the *Igf2r* imprinted domain in mice [20, 25, 26]. Although the lncRNA-based mechanisms for establishing *Igf2r* imprinted domain in mice are well documented, imprinting of *IGF2R* and its neighbouring genes in other eutherian mammals is different from that of mice. In cows, *IGF2R*, *AIRN*, *SLC22A2* and *SLC22A3* are imprinted in their placentas [33]. Although there is no information about imprinting of *SLC22A2* and *SLC22A3*, *IGF2R* is imprinted in sheep and dogs [34, 35]. In humans, genes of the *IGF2R* imprinted domain show an indicator of imprinting, but it is polymorphically imprinted in a subset of placentas [36]. In contrast, in pigs, the evidence is confused since in one study there is paternal *IGF2R* expression [37], in a second study there is maternal *IGF2R* expression [38] and in the third study there is bi-allelic expression [39]. *SLC22A3* is not imprinted in their placentas [40]. Therefore, while the *IGF2R* imprinted domain is likely to have been present in the common ancestor of eutherian mammals it may not have been strongly selected in some species such as pigs. Whether the *IGF2R* imprinted domain evolved in a marsupial ancestor or developed by convergent evolution in marsupial lineages is currently unknown. Comparing the *IGF2R* gene locus between marsupials and eutherians would clarify its evolution.

The marsupial *IGF2R* gene is also imprinted [41–43]. However, until recently, it was assumed that *IGF2R* in marsupials lacked key regulatory features such as *Airn* and a DMR [41, 43] as there was no DMR at intron 2, the location of the mouse *Igf2r* DMR/ICR. However, we described a novel DMR in intron 12 which has a 687 bp antisense lncRNA, *Antisense* *LncRNA* in *IGF2R*DMR (*ALID*) [43]. The DMR location is totally different between eutherians (intron 2) [26, 30] and marsupials (intron 12) [43]. *ALID* is much shorter than mouse *Airn* [43], so the imprinting mechanism of *IGF2R* in marsupials may be different from that of mouse *Igf2r*. However, it is still possible that *ALID* may have an analogous mode of action in silencing the flanking genes *SLC22A2/SLC22A3* in the placenta. If *SLC22A2* and/or *SLC22A3* are imprinted in the marsupial lineage, it would provide strong evidence that placenta-specific imprinting has been strongly selected for therian mammals, despite the differences in the DMR location.

In this study, the imprinting status of *SLC22A2* and *SLC22A3* in marsupial placentas was investigated using a marsupial, the tammar wallaby (*Macropus eugenii*). The tammar has an epithelio-chorial placenta which consists of two regions, the avascular bilaminar omphalopleure (BOM) and the vascular trilaminar omphalopleure (TOM) separated by the terminal blood vessel, the sinus terminalis [44]. Marsupial orthologues of *SLC22A2* and *SLC22A3* were investigated by combining molecular experiments and analysis of tammar transcriptome data sets. Imprinting analysis was thereafter performed in the tammar placentas and other tissues to confirm its imprinting. We further evaluated protein localisation to determine conserved transporter function. Here, we report that *SLC22A3* but not *SLC22A2* is imprinted in the tammar placenta. By confirming *SLC22A3* imprinting, this study revealed that the *IGF2R* imprinted domain has been strongly selected in marsupial mammals after either transposition or independent acquisition of the marsupial *IGF2R* DMR.

## Results

### *Identification of marsupial* SLC22A2

**Fig. 1:**
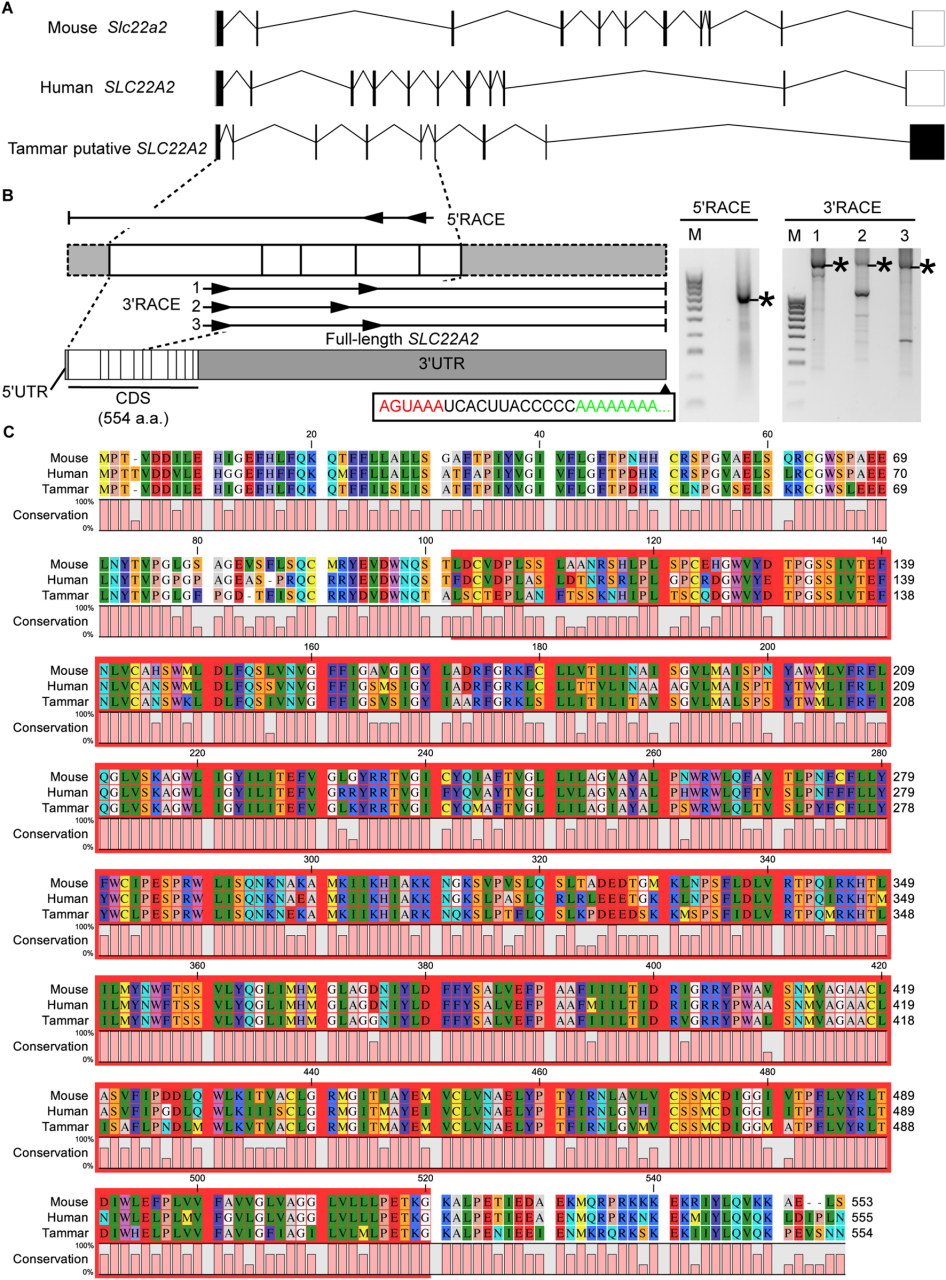
Identification of marsupial orthologue of *SLC22A2* in the tammar. **A** Exon structure of mouse *Slc22a2, human SLC22A2* and tammar putative *SLC22A2*. Black boxes and white boxes represent protein-coding exon and UTRs, respectively. **B** 5ʹ and 3ʹ RACE primers and RACE results. Full-length of tammar *SLC22A2* encoding 554 a.a. was determined by RACE experiments. Asterisks indicate RACE product containing partial *SLC22A2* sequences. 3ʹ RACE product contained poly-A signal (red-letters) and poly-A tail (green letters). **C** Protein alignment. Red-coloured highlight represents the major facilitator superfamily (MFS) domain of the tammar SLC22A2

To characterise the imprinting cluster of the marsupial *IGF2R* gene locus, the marsupial orthologue of *SLC22A2* was searched for using the wallaby genome database (Wallabase: https://wallabase.science.unimelb.edu.au/) comparing with mouse SLC22A2 (Accession number: NM_013667.3). 1693 bp of putative tammar *SLC22A2* were identified. The putative tammar *SLC22A2* was found in the vicinity of the tammar *IGF2R* gene. Furthermore, another SLC22A family gene, the *SLC22A1* gene candidate, was found between the putative *SLC22A2* and *IGF2R*, as seen in eutherians. Based on this conserved synteny, we considered the putative *SLC22A2* as a candidate gene for our downstream analysis. While mouse and human SLC22A2 have 11 exons including 5ʹ and 3ʹ UTRs, the putative tammar SLC22A2 had 9 exons without a 3ʹ UTR (Fig. 1A). To characterise potential isoforms of *SLC22A2* in placentas, full-length of tammar *SLC22A2* transcripts were examined by 5' and 3'. Rapid amplification of cDNA ends (RACE) reactions using BOM cDNA (Fig. 1B). While the 5ʹ RACE reaction produced a distinct single band, the 3' RACE reactions produced multiple bands depending on primers used (Fig. 1B). By comparing three different 3' RACE reactions, we confirmed that only the largest RACE product contained partial *SLC22A2* with a poly-A tail and upstream polyadenylation signals (Fig. 1B). The identified full-length tammar *SLC22A2* contained 11 exons and encoded 554 a.a (Fig. 1B). The identified tammar SLC22A2 had a high consensus with mouse Slc22a2 (similarity: 90%, identity: 79%) and human SLC22A2 (similarity: 88%, identity: 78%). The tammar SLC22A2 shared the major facilitator superfamily (MFS) domain with mouse Slc22a2 and human SLC22A2 (Fig. 1C).

### *The tammar* SLC22A2 *is not imprinted in placenta tissues*

**Fig. 2:**
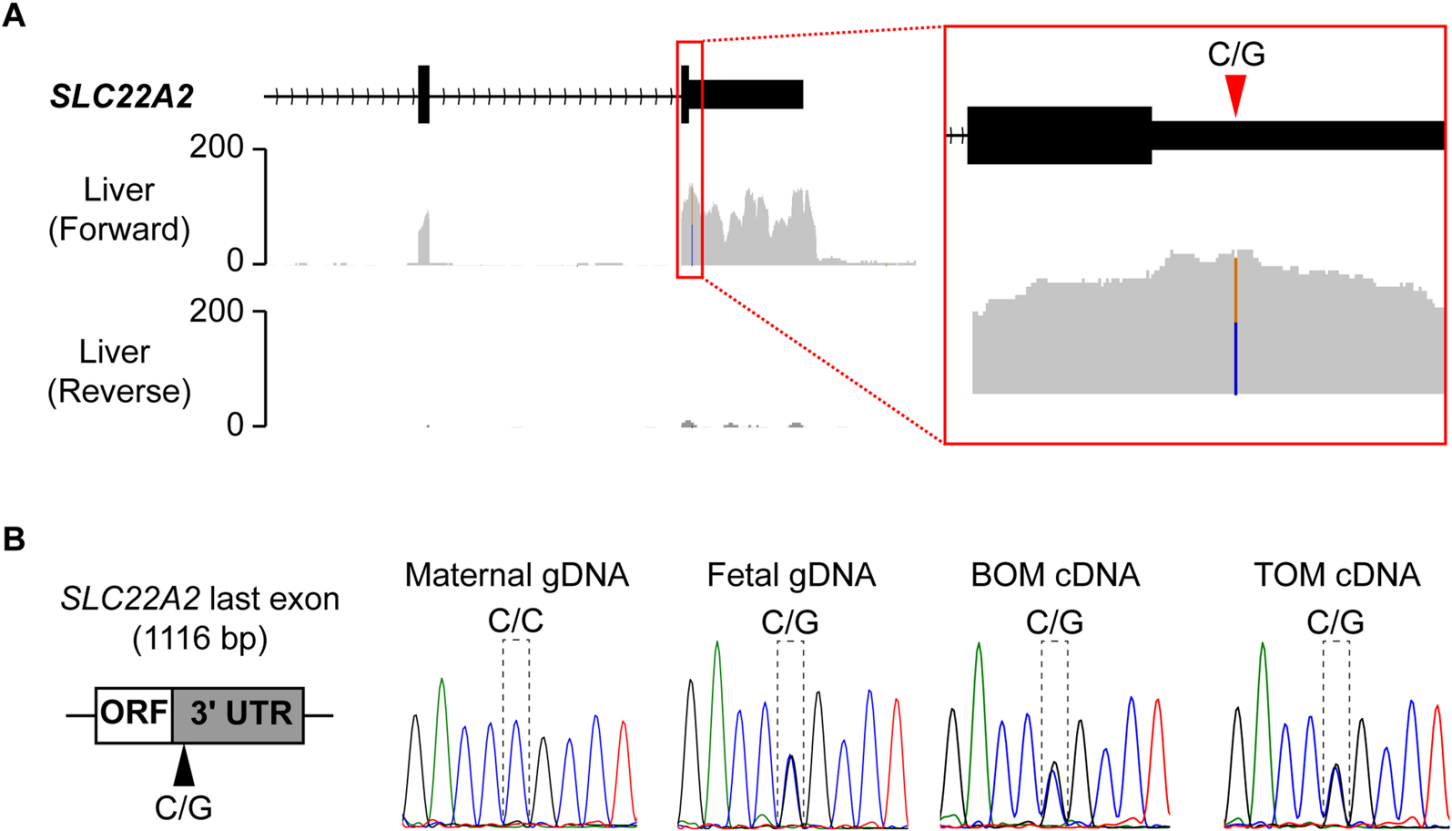
Tammar *SLC22A2* is not imprinted in placenta tissues*.* **A** Detecting a single nucleotide polymorphism (SNP) in tammar *SLC22A2* using liver transcriptome data*.* A SNP candidate (C/G) was detected at the 3'UTR of the last exon of tammar *SLC22A2*. The grey-coloured graph represents mapped RNA-seq reads. Black box with a line represents exon structure of the gene. **B** Allelic expression analysis of tammar *SLC22A2* in BOM and TOM tissues by direct sequencing followed by PCR amplification

In order to identify potential single nucleotide polymorphism (SNP) sites, publically available tammar transcriptome data sets were analysed (SRA accession number: DRP001145). After analysing several adult tissues (testis, liver, lung, heart, spleen and brain), transcriptome data derived from liver were confirmed to have mapped-reads to the *SLC22A2* exons which were confirmed by the RACE experiments. Fortunately, the liver transcriptome data had an informative SNP at the last exon of *SLC22A2* (Fig. 2A). The C/G SNP was further confirmed by PCR using fetal gDNA followed by direct sequencing of the PCR products. After confirming the heterozygous C/G SNP in fetal gDNA, allelic expression analysis of *SLC22A2* transcript was performed. In both BOM and TOM tissues, *SLC22A2* clearly showed bi-allelic expression (Fig. 2B).

### *Identification of marsupial* SLC22A3

**Fig. 3:**
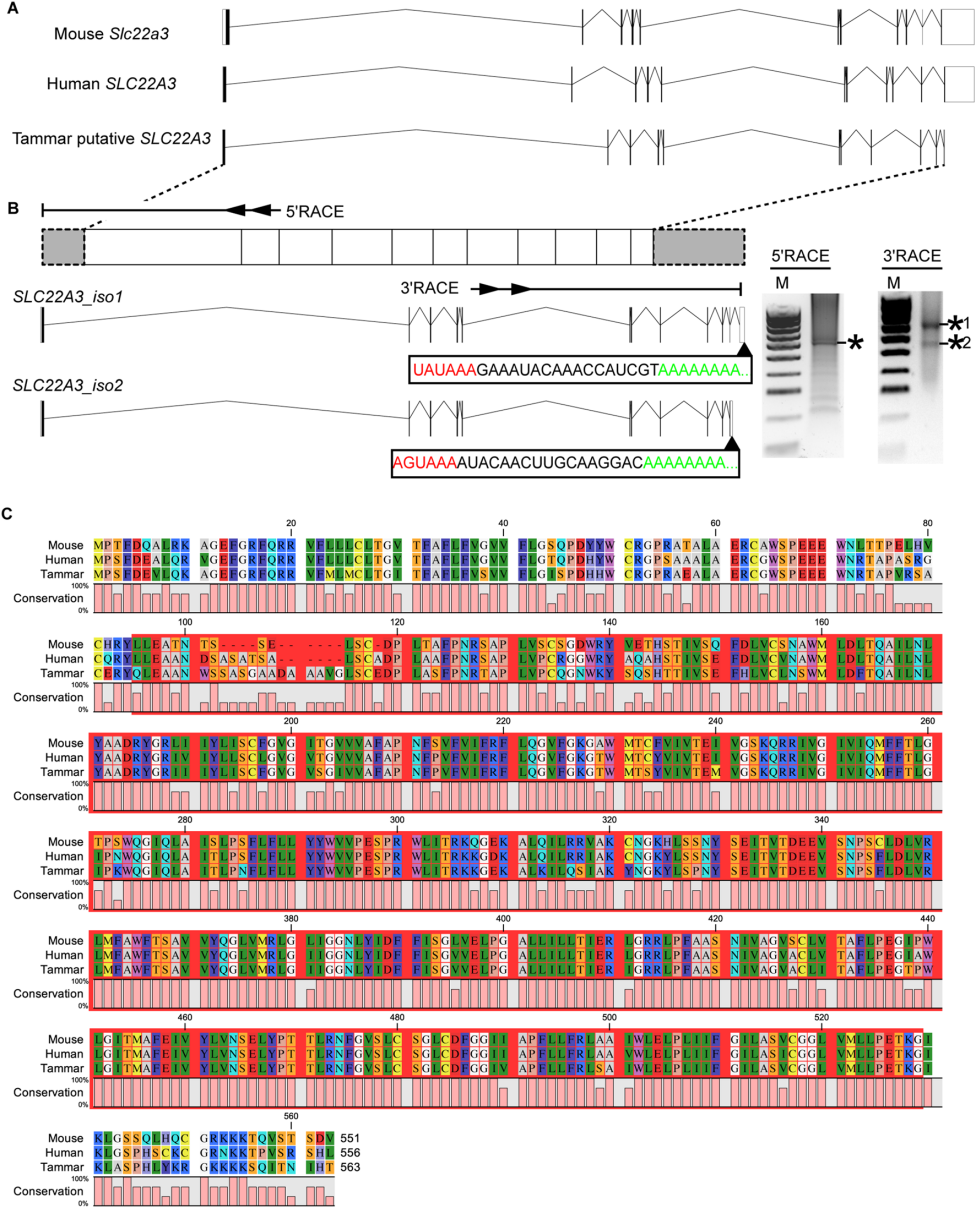
Identification of marsupial orthologue of *SLC22A3* in the tammar. **A** Exon structure of mouse *Slc22a3, human SLC22A3* and tammar putative *SLC22A3*. Black boxes and white boxes represent protein-coding exon and UTRs, respectively. **B** 5’ and 3’ RACE primers and RACE results. Two isoforms of tammar *SLC22A3* was determined by RACE experiments, and both encoded 563 a.a. Asterisks indicate RACE product containing partial *SLC22A3* sequences. 3ʹ RACE products contained poly-A signal (red-letters) and poly-A tail (green letters). **C** Protein alignment. Red-coloured highlight represents the major facilitator superfamily (MFS) domain of the tammar SLC22A3

To characterise the imprinting cluster of marsupial *IGF2R* gene locus, marsupial orthologue of *SLC22A3* was searched for using the wallaby genome database (Wallabase: https://wallabase.science.unimelb.edu.au/) with mouse SLC22A3 (Accession number: NM_011395.2). 1692 bp of putative tammar *SLC22A3* were identified. The putative tammar *SLC22A3* was found next to the tammar *SLC22A2* gene, as seen in eutherians. Based on this conserved synteny, we considered the putative *SLC22A3* as a candidate gene for our downstream analysis. While mouse and human *SLC22A3* have 11 exons including 5ʹ and 3’ʹ UTRs, the putative tammar SLC22A3 had 11 exons without the 3ʹ UTR (Fig. 3A). To characterise potential isoforms of *SLC22A3* in placentas, full-length of tammar *SLC22A3* transcripts were examined by 5' and 3' RACE reactions using BOM cDNA (Fig. 3B). While the 5’RACE reaction produced a distinct single band, the 3' RACE reactions produced two different RACE products (Fig. 3B). After cloning and sequencing each RACE product, two different isoforms were confirmed. Both isoforms had poly-A tail and upstream polyadenylation signals (Fig. 3B). Although there were at least two isoforms based on the differences in the exon structure, all isoforms encoded the same 563 amino acids. The identified tammar SLC22A3 had a high homology with mouse Slc22a3 (similarity: 89%, identity: 81%) and human SLC22A3 (similarity: 91%, identity: 84%). The tammar SLC22A3 shared the MFS domain with mouse and human SLC22A3 (Fig. 3B and C).

### *The tammar* SLC22A3 *is imprinted in bilaminar placenta tissues*

**Fig. 4:**
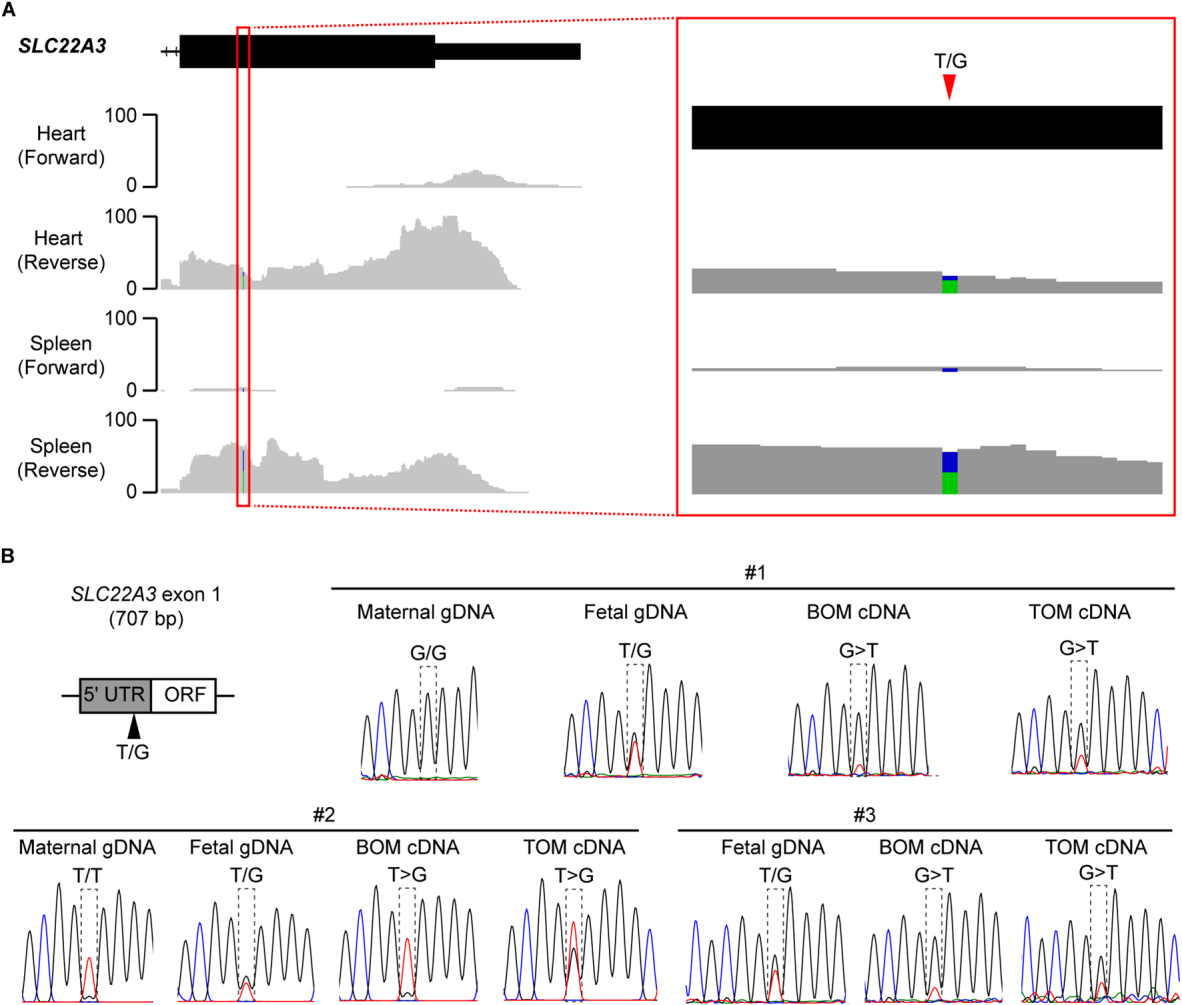
Tammar *SLC22A3* is imprinted in BOM tissues. **A** Detecting a single nucleotide polymorphism (SNP) in tammar *SLC22A3* using heart and spleen transcriptome data*.* A SNP candidate (T/G) was detected at the first exon of tammar *SLC22A3*. The grey-coloured graph represents mapped RNA-seq reads. Black box with a line represents exon structure of the gene. **B** Allelic expression analysis of tammar *SLC22A3* by direct sequencing followed by PCR amplification. Animal#1 and Animal#2 showed clear maternal expression in BOM tissues

In order to identify potential SNP sites, published tammar transcriptome data sets were analysed. After analysing several adult tissues (testis, liver, lung, heart, spleen and brain), transcriptome data sets derived from heart and spleen were confirmed to have mapped-reads matched the *SLC22A3* exons based on the RACE experiments. Fortunately, both heart and spleen transcriptome data had an informative SNP at the first exon of *SLC22A3* (Fig. 4A). After confirming the T/G SNP by PCR reaction, allelic expression analysis of *SLC22A3* transcripts was performed using the SNP information. Of the 20 biological replicates examined in this study, 3 samples had a clear heterozygous SNP. In BOM tissues (*n* = 3), *SLC22A3* showed strongly skewed allelic expression with the signal intensities that differed between the two alleles more than fivefold and 2 out of the 3 animals showed clear maternal expression as the mothers had homozygous SNP (Fig. 4B). The other animal also showed a strongly skewed allelic expression (Fig. 4B). However, since the mother of the animal was heterozygous, we could not conclude its parental origin-specific expression. The trilaminar placenta tissues of the same animals did not show the same strongly biased allelic expression as seen in the BOM tissue (Fig. 4C).

### SLC22A3 *has variable expression in pouch young heart and spleen*

**Fig. 5:**
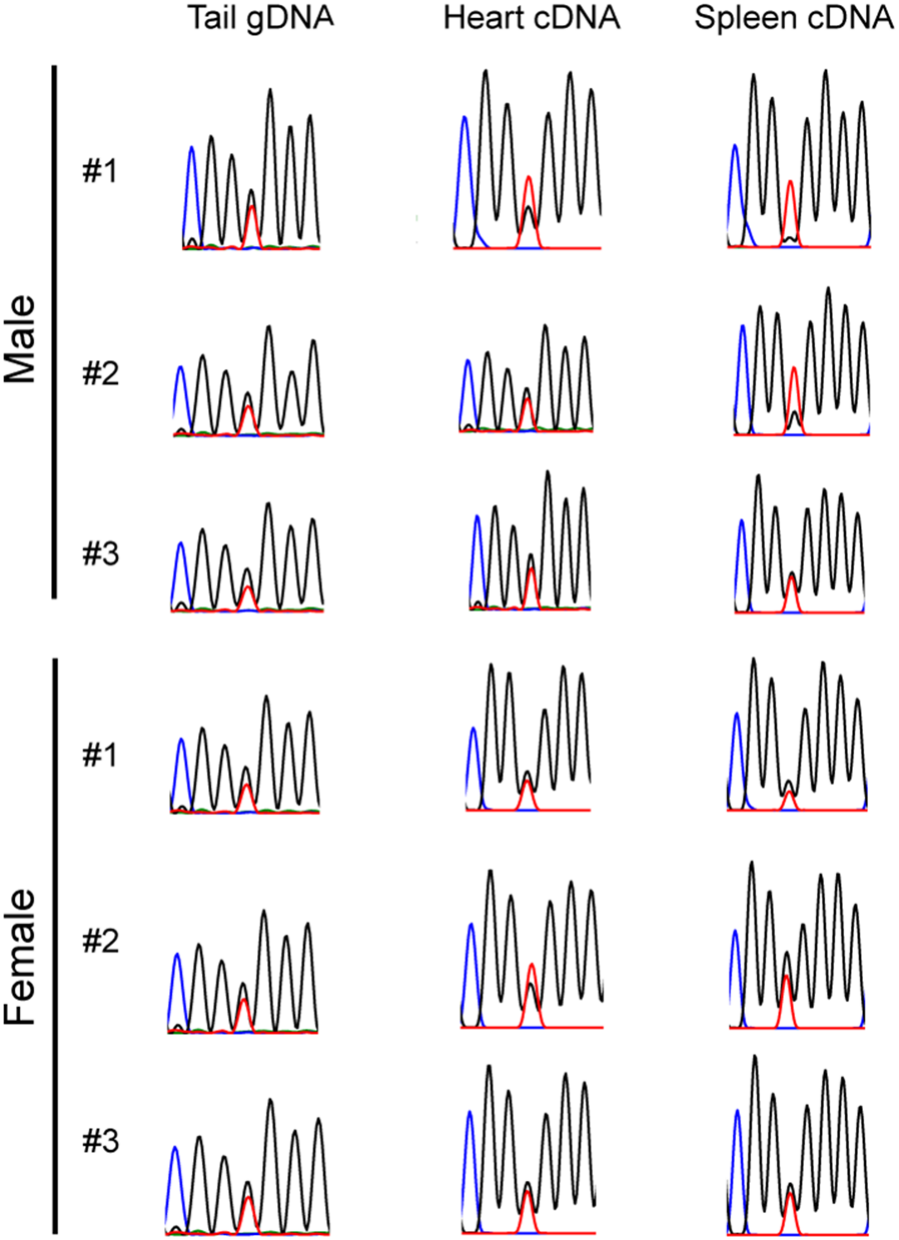
Biallelic expression of *SLC22A3* in the tammar heart and spleen. Allelic expression analysis of tammar *SLC22A3* in heart and spleen was examined in 6 PYs (3 males and 3 females) by direct sequencing followed by PCR amplification. In heart, all animals showed bi-allelic expression of *SLC22A3*. However, in spleen, two males had skewed expression while the other male and females showed bi-allelic expression of *SLC22A3*

Since mouse *Slc22a3* imprinting is only observed in the placenta [20], the tammar *SLC22A3* may have a similar tissue-specific imprinting pattern. To confirm tissue-specific imprinting of *SLC22A3* in the tammar, allelic expression of *SLC22A3* was investigated using the tammar pouch young (PYs). Heart and spleen tissues derived from PYs were used for this analysis as the transcriptome data showed *SLC22A3* expression in those tissues. After checking 12 animals, 6 animals had the heterozygous T/G SNP. In all of the 6 animals, *SLC22A3* showed bi-allelic expression in heart (Fig. 5). In spleen, while two males had skewed expression with signal intensities that differed between the two alleles more than twofold, the other males and the three females showed bi-allelic expression (Fig. 5).

### SLC22A3 *imprinting is not promoter DNA methylation dependent*

**Fig. 6:**
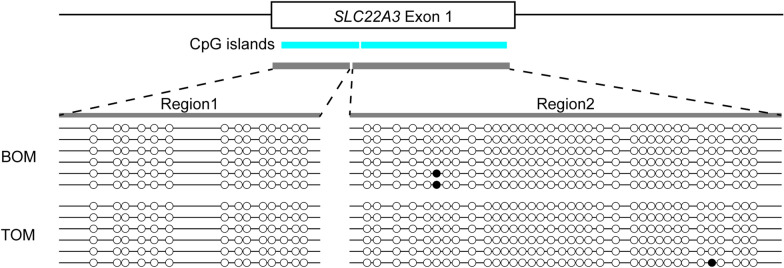
Tammar *SLC22A3* lacked promoter DNA methylation. DNA methylation of the putative promoter of *SLC22A3* in the tammar BOM and TOM placenta. Aqua-coloured region represent CpG islands determined by MethPrimer programme. DNA methylation of the two CpG islands were examined by Bisulphite sequencing. Filled and opened circle represent methylated and unmethylated cytosine residue, respectively

Given the imprinting of tammar *SLC22A3* in the tammar placenta, we next analysed DNA methylation at its promoter to ask whether tammar *SLC22A3* imprinting is DMR dependent or not. Based on the MethPrimer programme, we identified two CpG islands located over the putative promoter region of the tammar *SLC22A3* (Fig. 6). Bisulphite sequencing of the CpG islands demonstrated that the majority of CpG sites were unmethylated in the entire region of the putative *SLC22A3* promoter in both BOM and TOM tissues (Fig. 6).

### SLC22A3 is present in endodermal cells of the BOM and TOM

**Fig. 7:**
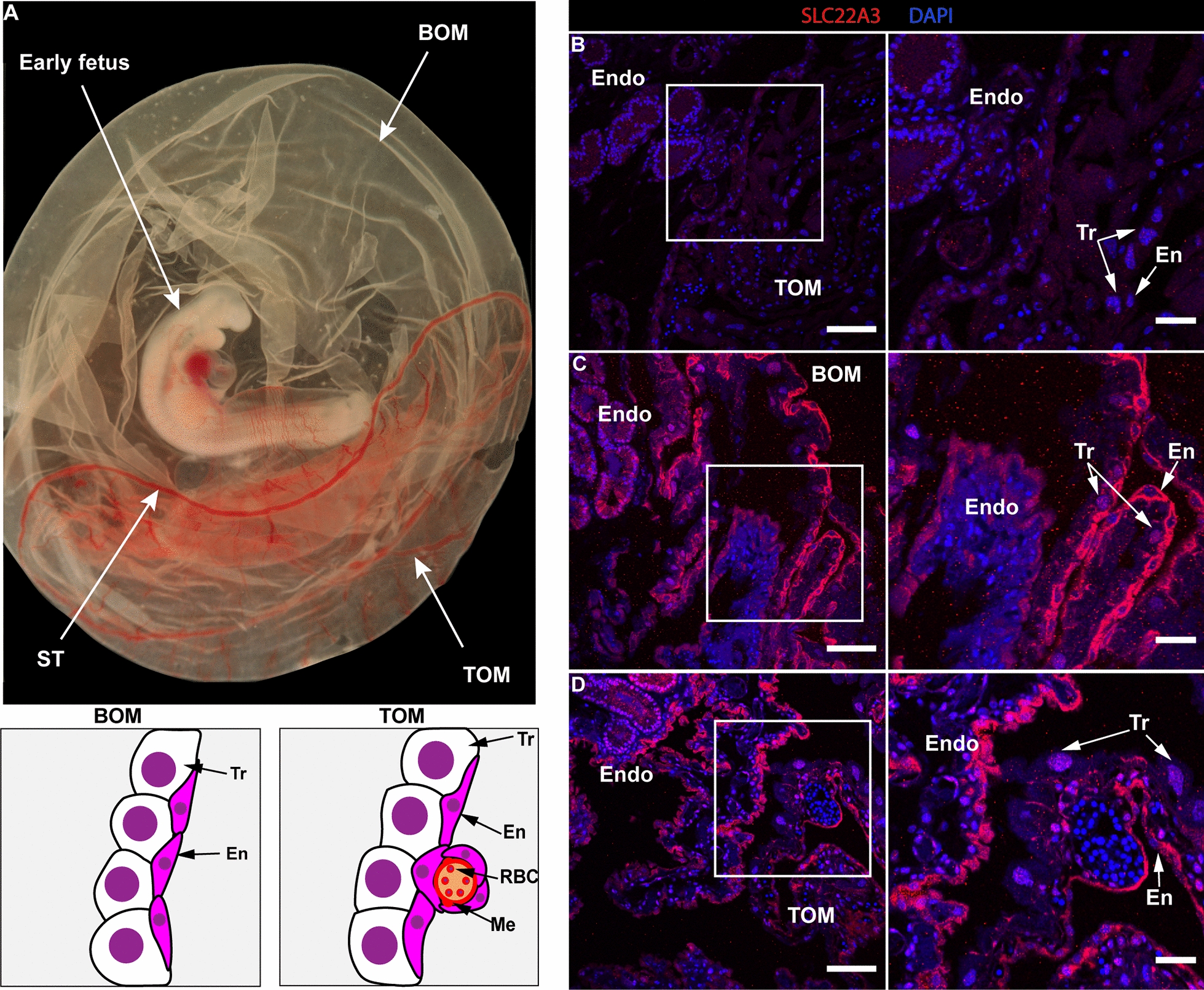
Tammar SLC22A3 localised at the placental endodermal cells. **A** An example of the early tammar fetus and placenta. Schematic diagram below the photo represents tammar placental structure and placental cell types. While the bilaminar omphalopleure (BOM) is the non-vascularised part of placenta, the trilaminar omphalopleure (TOM) is the vascularised part of placenta. The sinus terminalis (ST) is the blood vessel that marks the boundary between the vascular and non-vascular regions, BOM and TOM contains trophoblast cells (Tr) and endodermal cells (En). The mesodermal (Me) layer of TOM has red blood cells (RBC) in the developing blood vessels. **B** Immunofluorescence with IgG negative control. **C** Immunofluorescence of SLC22A3 in BOM placenta tissue. **D** Immunofluorescence of SLC22A3 in TOM placenta tissue. Red and blue colours represent SLC22A3 and DAPI staining, respectively. Right-hand side panels show high power photos. Scale bars: 100 μm and 40 μm (high power and low power) panels. Endo: endometrium

To determine its conserved role in placental tissues, protein localisation of SLC22A3 in the BOM and TOM was examined (Fig. 7). SLC22A3 was localised at the luminal surface of endometrial tissues (Fig. 7B). SLC22A3 protein was detected in the endodermal cells of both BOM and TOM placental tissues (Fig. 7C).

## Discussion

In the tammar placentas, *SLC22A3* but not *SLC22A2* was imprinted. As in the mouse, tammar *SLC22A3* imprinting was evident in BOM placenta tissues but not in the other tissues examined in this study. We conclude that the *SLC22A3* imprinting in mammalian placentas evolved within the *IGF2R* imprinted domain in both marsupial and eutherian mammals.

SLC22A3 is a poly-specific organic cation transporter that transfers a wide variety of substrates and toxins across the cell membrane [45–47]. In *Slc22a3* knockout mice, although there are no obvious placental and fetal growth defects, there are effects on placental transfer functions [48]. In concert with its transporter activity, mouse *Slc22a3* is localised in the labyrinth layer of its chorio-allantoic placenta [49–51] where nutrient transfer from maternal blood occurs [52]. Mouse *Slc22a3* is also present in the visceral endoderm of the yolk sac [51], where nutrient uptake into the embryo occurs [53]. In the tammar chorio-vitelline placenta, BOM appears to be responsible for a greater uptake of nutrients from uterine secretions than TOM [54–57]. *SLC22A3* imprinting was evident in BOM tissues, suggesting that *SLC22A3* imprinting is associated with uptake of nutrients from uterine secretions by the avascular BOM. Based on immunofluorescence, we confirmed that the tammar SLC22A3 is localised in the endodermal cell layer of both BOM and TOM side of the tammar yolk sac (chorio-vitelline) placenta. The tammar placental endodermal cells are also derived from trophoblast, and in this and the mouse study [51] SLC22A3 expression in the endoderm cells of the yolk sac is conserved between mouse and the tammar. Furthermore, since a labyrinth layer marker, GCM1, is also present in the endodermal cell layer of the tammar placenta [56, 58], our data suggest that SLC22A3 is a conserved nutrient transporter in therian placentas and supports the previous suggestions [56, 59] that the endodermal cell layer functions as a centre of nutrient trafficking in the tammar placenta.

In the tammar placenta, the *SLC22A3* imprinting was evident only in BOM tissues. However, it is currently unknown why only BOM tissues but not TOM tissues show imprinted *SLC22A3* expression in the tammar placenta. In concert with their functional role in either nutrient transport (BOM) or respiration (TOM) [44, 57], BOM and TOM have different transcriptional profiles [56]. This suggests that BOM tissues are differentially transcriptionally regulated, resulting in imprinted expression of *SLC22A3*. Alternatively, other cells such as mesodermal cells and nucleated red blood cells in TOM tissues may express the *SLC22A3* gene. In mice, *Slc22a3* is highly expressed in the visceral endoderm and is present, albeit at low expression, in the mesoderm cells of the visceral yolk sac [51], so, it is possible that mesoderm cells masks *SLC22A3* imprinted expression in the tammar TOM.

The mouse *Igf2r* imprinted domain is regulated by the lncRNA, *Airn* [20, 28, 31]. The *Airn* transcript recruits a histone modification enzyme, EHMT2, and adds an inactive histone-3 lysine-9 di-methylation at the *Slc22a3* gene locus [31]. In addition, *Airn* recruits H3K27me3 to *Slc22a2* and *Slc22a3* to establish the broad imprinted domain in mice [22], and there is no promoter DNA methylation at either *Slc22a2* or *Slc22a3* in mice [20, 51], suggesting that both are regulated by histone modification-based imprinting. Similarly, human *SLC22A2* and *SLC22A3* also lack a promoter DMR in placenta [36]. Like the tammar, the cow has a chorio-epithelial placenta, and has *SLC22A3* but not *SLC22A2* with a DMR at the promoter region in its placenta [33]. Since *AIRN* is conserved across eutherians [33, 60], the bovine study indicates that *SLC22A3* imprinting mechanism can be varied even within eutherian mammals. In our analysis, CpG islands at the putative promoter of the tammar *SLC22A3* lacked DNA methylation. This suggests that tammar *SLC22A3* imprinting is not directly silenced by a DMR on its promoter as in mice and humans. In this context, the tammar lncRNA, *ALID*, is likely to have a similar function to *Airn* as this lncRNA is also expressed from a DMR which may be an ICR [43]. Further analysis of the interactions between EHMT2, PRC2 and *ALID* would be interesting in subsequent studies to determine potential function of *ALID* in silencing *SLC22A3* in the tammar placenta.

**Fig. 8:**
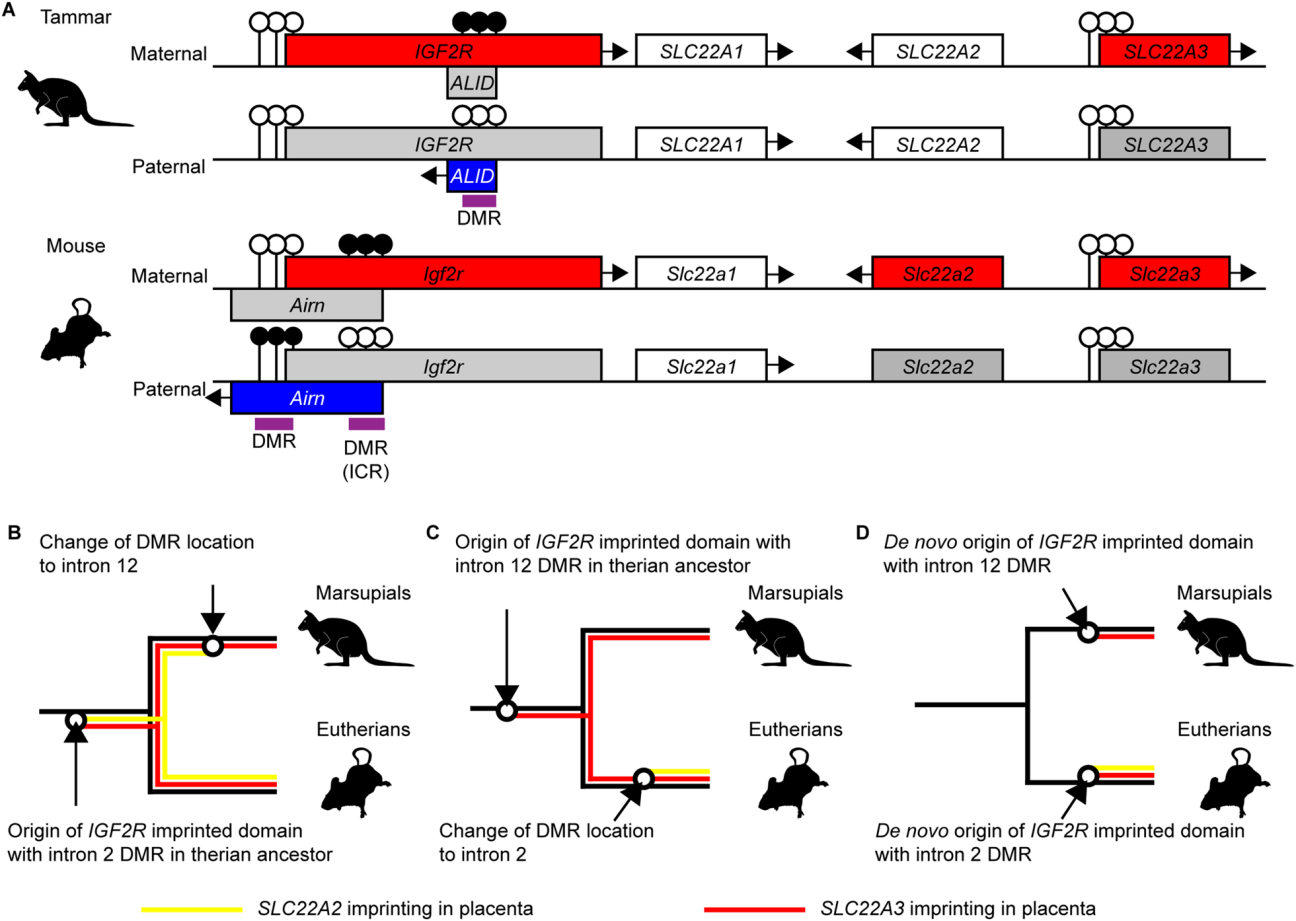
The evolution of the *IGF2R* imprinted domain and the intragenic DMR. **A** Schematic diagram of the tammar *IGF2R* imprinted domain and mouse *Igf2r* imprinted domain. The *IGF2R* DMR is located in intron 12 in the tammar whereas the eutherian *IGF2R* DMR is located in the promoter and intron 2. Red-coloured boxes and blue-coloured boxes represent maternally expressed genes and paternally expressed genes, respectively. White and grey-coloured boxes represent non-imprinted and silenced genes, respectively. Arrows indicate the direction of transcription. There are three scenarios for the evolution of *IGF2R* DMR and *SLC22A3* imprinting in therian mammals. **A** Transposition of the *IGF2R* DMR in marsupials. In this case, the *IGF2R* DMR evolved in the common ancestor of therian mammals and the DMR acts as an ICR to establish epigenetic silencing of *SLC22A2* and *SLC22A3* as in mice. Even after the transposition, the *SLC22A3* imprinting but not *SLC22A2* was positively maintained in marsupial lineage. **B** Transposition of the *IGF2R* DMR in eutherians. In this case, the *IGF2R* DMR evolved in the common ancestor of therian mammals and the DMR acts as an ICR to establish epigenetic silencing of *SLC22A3* as in the tammar. After the transposition, the *SLC22A2* imprinting evolved in eutherian lineage. **C** Independent acquisition of the *IGF2R* DMR in each mammalian lineage. In this case, the evolution of *SLC22A3* imprinting would have accompanied the de novo acquisition of *IGF2R* DMR in each mammalian lineage. Yellow and red lines indicate that *SLC22A2* imprinting and *SLC22A3* imprinting, respectively

In marsupials, the *IGF2R* DMR is located in intron 12 while the eutherian *IGF2R* DMR is located in intron 2 [26, 43, 61] (Fig. 8A). Based on this difference in genomic position, marsupial and eutherian *IGF2R* DMRs may have been translocated after the divergence of marsupials and eutherians (Fig. 8B and C), or they may have been acquired independently in each mammalian lineage after the marsupial–eutherian split (Fig. 8D) [43]. In either case, our study demonstrates that the *SLC22A3* imprinting in placentas has been strongly selected in therian mammals during mammalian evolution (Fig. 8). Since SLC22A3 is a poly-specific transporter in placental tissues that regulates placental transfer, this selection may have occurred to control the balance between supply and demand of nutrients.

## Conclusions

By confirming imprinting of the *SLC22A3* gene in a marsupial placenta, our data suggest the *SLC22A3* imprinting has been strongly selected in both marsupials and eutherians the differences in the DMR locations. Our data further suggest that the control of nutrient transport in the placenta is a critical evolutionary function of genomic imprinting in marsupial, as well as in eutherian placentas.

## Material and methods

### Animals

Tammar wallabies (*Macropus eugenii*), of Kangaroo Island origin, were held in open grassy yards in our breeding colony at the University of Melbourne. Adults and pouch young (PY) were killed humanely as previously described [14, 55, 57]. Placentas from fetuses in the final third of gestation [day 19–25 days of the 26.5 day pregnancy (*n* = 20)] were collected post mortem from adult females and either snap frozen or fixed in 4% (w/v) paraformaldehyde (PFA) in phosphate buffered saline (PBS) (140 mM NaCl, 2.7 mM KCl, 10 mM Na_2_HPO_4_, 1.8 mM KH_2_PO_4_, pH7.4), washed and stored in 100% methanol before histological analysis. PY aged between day 33 and day 81 after birth (*n* = 12) were dissected post mortem and heart and spleen snap frozen immediately. All animal experiments were approved by the University of Melbourne Animal Experimental Ethics Committees and followed the Australian National Health and Medical Research Council (2013) guidelines.

### RNA extraction and cDNA synthesis

Snap-frozen placenta tissues consisting of the separated avascular bilaminar omphalopleure (BOM) and the vascular trilaminar omphalopleure (TOM) (see Fig. 7A), PY spleen and PY heart were used for RNA extraction using the GenElute Mammalian total RNA Miniprep Kit (Sigma-Aldrich, Missouri, USA) following the manufacturer’s instructions. The extracted RNA was treated with the DNA-free DNase treatment and removal kit (Thermo Fisher Scientific, Massachusetts, USA) to remove residual genomic DNA (gDNA). After confirming removal of gDNA by a Nanodrop (Thermo Fisher Scientific, Massachusetts, USA) and PCR using RNA as a template, either 200 ng of placenta RNA or up to 800 ng of PY spleen RNA or up to 400 ng of PY heart RNA were used as templates for cDNA synthesis using SuperScript IV First strand Synthesis System (Invitrogen, Carlsbad, USA).

### 5' and 3' rapid amplification of cDNA ends (RACE)

To determine the complete full sequence of marsupial *SLC22A2* and *SLC22A3*, 5' and 3', rapid amplification of cDNA ends (RACE) experiments were performed using a SMARTer RACE 5'/3' kit (Clontech, California, USA). The first RACE reactions were performed with BOM cDNA using SeqAmp DNA Polymerase (Clontech, California, USA) with gene specific primers (Additional file 1). The nested 5' and 3' RACE reactions were performed by GoTaq DNA polymerase (Promega, Wisconsin, USA) and the RACE products were cloned using pGEM-T Easy Vector (Promega, Wisconsin, USA) and *Escherichia coli* JM109 competent cells (Promega, Wisconsin, USA). Plasmids were extracted using Wizard Plus SV Minipreps DNA Purification System (Promega, Wisconsin, USA) and directly sequenced with M13 primers (Additional file 1).

### *Comparative analysis of therian* SLC22A2 *and* SLC22A3

﻿DNA sequences of human *SLC22A2*, human *SLC22A3*, mouse *Slc22a2* and mouse *Slc22a3* were obtained from NCBI (https://www.ncbi.nlm.nih.gov). Amino acid sequences retrieved from DDBJ/EMBL/GenBank/RefSeq database were used for generating alignment using CLC sequence viewer 8 (https://resources.qiagenbioinformatics.com/manuals/clcsequenceviewer/current/index.php?manual=CLC_Sequence_Viewer_vs_Workbenches.html). Accession numbers: *Homo sapiens SLC22A2*, NM_003058.4; *Mus musculus Slc22a2*, NM_013667.3; *H. sapiens SLC22A3*, NM_021977.4; *M. musculus Slc22a3*, NM_011395.2. Functional domains of deduced amino acid sequences were examined with the Prosite server (http://prosite.expasy.org/).

### Transcriptome analysis

To characterise the *IGF2R* imprinted domain as well as identifying informative single nucleotide polymorphisms (SNPs), tammar transcriptome data sets derived from various tissues (testis, liver, lung, heart, spleen and brain) were analysed. Publicly available tammar raw RNA-seq data sets (DRP001145) were downloaded from NCBI SRA (https://www.ncbi.nlm.nih.gov/sra). All RNA-seq reads were trimmed using TrimGalore! (v0.6.5) (https://github.com/FelixKrueger/TrimGalore) with default settings. The trimmed reads were aligned to the wallaby genome.v3 (https://wallabase.science.unimelb.edu.au) using HISAT2 (v2.1.0) [62] with a parameter—rna-strandness FR to reflect the strandedness of sequenced RNA. The mapped reads were assigned to each strand by Samtools (v1.9) [63]. SNP sites were called using BCFtools (v1.9) and the output file was compared with the mapped reads on Integrative genome viewer (IGV) [64, 65].

### Genomic DNA extraction

Snap-frozen BOM, TOM, PY tails and endometrial tissues were used for genomic DNA (gDNA) extraction. BOM and TOM were the fetal gDNA source and endometrium was the maternal gDNA source. PY tails were used for genotyping. DNA extraction was performed with Wizard Genomic DNA purification kit (Promega, Wisconsin, USA) following the manufacturer’s instructions.

### Allelic expression analysis

Extracted gDNA was used as a template for PCR amplification. PCR reaction was performed using gene specific primers (Additional file 1) with Go-Taq polymerase (Promega, Wisconsin, USA) under the following cycle conditions: 95 °C 30 s, 65 °C 30 s, and 72 °C 1 min. To analyse sequences of the tammar *SLC22A2* and *SLC22A3 transcripts*, strand-specific cDNA synthesis was performed using each gene specific reverse primer (Additional file 1). The synthesised cDNA was used as a template for PCR amplification with Go-Taq polymerase (Promega, Wisconsin, USA) under the following cycle conditions: 95 °C 30 s, 65 °C 30 s, and 72 °C 1 min. After performing gel electrophoresis, confirmed PCR products from gDNA and cDNA were extracted and directly sequenced by Sanger sequencing to confirm SNP sites and allele specific expression. We consider a biased expression as a strongly skewed expression when the signal intensities between the two alleles differed more than fivefold.

### Bisulphite sequencing

Purified genomic DNA derived from the fetus used for allelic expression analysis was treated with sodium bisulphite solution using EpiMark Bisulfite Conversion kit (New England Biolabs, Massachusetts, USA). After the bisulphite treatment of the genomic DNA, 40 cycles of PCR were carried out using EpiTaq polymerase (Takara Bio, Shiga, Japan) with the primers (Additional file 1) designed by MethPrimer (https://www.urogene.org/methprimer/) [66]. The PCR products were cloned using a pGEM T-easy vector (Promega, Wisconsin, USA) and *E. coli* JM109 competent cells (Promega, Wisconsin, USA). Plasmids were purified using Wizard Plus SV Minipreps DNA Purification System (Promega, Wisconsin, USA) and directly sequenced using M13 primers (Additional file 1). The sequence data were analysed by quantification tool for methylation analysis (QUMA) programme (http://quma.cdb.riken.jp) [67].

### Immunofluorescence (IF) staining

PFA-fixed placenta samples (BOM and TOM at day 22 of gestation) were washed in 1 × PBS and re-hydrated through an ethanol series before being embedded in paraffin. Embedded samples were serially sectioned at 5 μm and mounted on poly-lysine coated slides (ThermoFisher Scientific, Massachusetts, USA). The sections were de-waxed, rehydrated through decreasing concentrations of ethanol, and then incubated in 0.1% (v/v) Triton X-100 in 1 × PBS (PBST) for 15 min at room temperature to permeabilise the tissue. Slides were boiled in Tris–EDTA (pH 8.0) for 20 min. The sections were treated with 0.3% (w/v) Sudan Black in 70% (v/v) EtOH solution for 15 min to reduce auto-fluorescent background. The Sudan Black treated sections were washed by 70% (v/v) EtOH and 1 × PBS before blocking. Thereafter, the sections were incubated for 1 h with 10% (w/v) goat serum diluted in 1 × PBS. After blocking, sections were incubated with primary antibody solution (Additional file 2) at 4 °C for 16 h. The following day, sections were washed three times with PBS and then incubated with fluorescent secondary antibodies (Additional file 2) for 1 h. The sections were washed three times with 1 × PBS again and then incubated for 10 min with 4', 6-diamidino-2-phenylindole (DAPI) (Sigma-Aldrich, Missouri, USA). DAPI-treated sections were mounted with fluorescence mounting solution. The controls for all treatments were no primary antibody and IgG isotype control antibodies. Images were collected on a Nikon A1R Confocal Laser Microscope System (Nikon, Tokyo, Japan).

## Supplementary Information

**Additional file 1. **Primers used for this study.

**Additional file 2. **Antibodies used for this study.

## Data Availability

The datasets analysed during the current study are available in the NCBI SRA (https://www.ncbi.nlm.nih.gov/sra) as DRP001145.

## Abbreviations

DMR: Differentially methylated region
SLC22A2: Solute carrier family 22 member 2
SLC22A3: Solute carrier family 22 member 3
ICR: Imprinting control region
IGF2R: Insulin-like growth factor 2 receptor
lnc: Long non-coding
H3K27me3: Histone 3 lysine 27 trimethylation
PRC2: Polycomb repressive complex 2
ALID: Antisense lncRNA in IGF2R DMR
BOM: Bilaminar omphalopleure
TOM: Trilaminar omphalopleure
RACE: Rapid amplification of cDNA ends
a.a: Amino acids
