# Supplementary material for: Placental imprinting of SLC22A3 in the IGF2R imprinted domain is conserved in therian mammals
Source: Epigenetics Chromatin. 2022 Aug 27;15:32. doi: 10.1186/s13072-022-00465-4 (PMC9419357; doi:10.1186/s13072-022-00465-4)
Supplement: Supplementary file 1 — Additional file 1. Primers used for this study. [file 13072_2022_465_MOESM1_ESM.docx]

**Additional file 1. Primers used for this study**

| Primer | Sequence |
| --- | --- |
| 5'/3' Race reaction |  |
| *SLC22A2* |  |
| 5'RACE | 5'-TGGGAGACTCTGGAAGGCACCAATA-3' |
| 5'RACE nested | 5'-ATTCCCACAGTCCTCCGGTACTTCA-3' |
| 3'RACE | 5'-AGCTGCACAGAACCCTTAGCCAATT-3' |
| 3'RACE nested_1 | 5'-GAATTTGTCGGCCTGAAGTACCGGA-3' |
| 3'RACE nested_2 | 5'-TTATCCAAGGACTGGTCAGCAAGGC-3' |
| 3'RACE nested_3 | 5'-CGGAGGACTGTGGGAATTTGCTACC-3' |
| *SLC22A3* |  |
| 5'RACE | 5'-ATAGCACAGAGTAAAAGCCCCAGCC-3' |
| 5'RACE nested | 5'-CCAGGTGAAACTCACTGACAATGGTGG-3' |
| 3'RACE | 5'-TTTACCAAGGACTTGTCATGCGCCT-3' |
| 3'RACE nested | 5'-GAAGGGACACCATGGTTGAGAACCT-3' |
| Sequencing |  |
| M13 forward | 5'-GTAAAACGACGGCCAGT-3' |
| M13 reverse | 5'-CAGGAAACAGCTATGAC-3' |
| Allelic expression |  |
| *SLC22A2* |  |
| Strand-specific reverse-transcription | 5'-AAGCTGTTGTGTTTCTTCCCACCCT-3' |
| Forward | 5'-CATGCCTGGGTAGAATGGGGATCAC-3' |
| Reverse | 5'-CAGGATGTAGCACCCTAGGCCTTTG-3' |
| *SLC22A3* (gDNA) |  |
| Forward | 5'-GCAGGGTATCTCCAGTCATTGACCA-3' |
| Reverse | 5'-GGCCAACAGTATATCGGGTGAGTCC-3' |
| *SLC22A3* (cDNA) |  |
| Strand-specific reverse-transcription | 5'-GGAGCAAATGCCACAACGATACCAC-3' |
| Forward | 5'-CACTTTTGCCTTCCTCTTCGTCAGC-3' |
| Reverse | 5'-ATAGCACAGAGTAAAAGCCCCAGCC-3' |
| Bisulphite sequencing |  |
| Region1 |  |
| Forward | 5'-GTAGGAAAGTGTTTTGGGGTAGTAG-3' |
| Reverse | 5'-CAAACACATCAACATAAACACCC-3' |
| Region2 |  |
| Forward | 5'-GGGTATTATTTTTGTTTTTTTT-3' |
| Reverse | 5'-AAAACTAACTATACTTCCAATTACCCTAAC-3' |
