# Supplementary material for: Placental imprinting of SLC22A3 in the IGF2R imprinted domain is conserved in therian mammals
Source: Epigenetics Chromatin. 2022 Aug 27;15:32. doi: 10.1186/s13072-022-00465-4 (PMC9419357; doi:10.1186/s13072-022-00465-4)
Supplement: Supplementary file 2 — Additional file 2. Antibodies used for this study. [file 13072_2022_465_MOESM2_ESM.docx]

**Additional file 2. Antibodies used for this study**

| Target | Host | Product code | Concentration (mg/ml) | Dilution |
| --- | --- | --- | --- | --- |
| Primary antibody | | | | |
| Human SLC22A3 | Rabbit | ab183071 | 0.50 | 1/250 |
|  |  |  |  |  |
| Secondary antibody | | | | |
| Rabbit immunogen | Goat | Alexa Fluor 568 | 2 | 1/500 |
